# Supplementary material for: Conditional chemoconnectomics (cCCTomics) as a strategy for efficient and conditional targeting of chemical transmission
Source: eLife. 2024 Apr 30;12:RP91927. doi: 10.7554/eLife.91927 (PMC11060718; doi:10.7554/eLife.91927)
Supplement: Figure 3—source data 3. [file elife-91927-fig3-data3.zip › Figure 3 source data3 part 1.zip!/Figure 3-Source data 3-part1/1071-1-CRISPResso_Report_osfbjvga.zip!/CRISPResso_on_1071_3/4c.Quantification_window_insertion_deletion_substitution_locations.pdf]

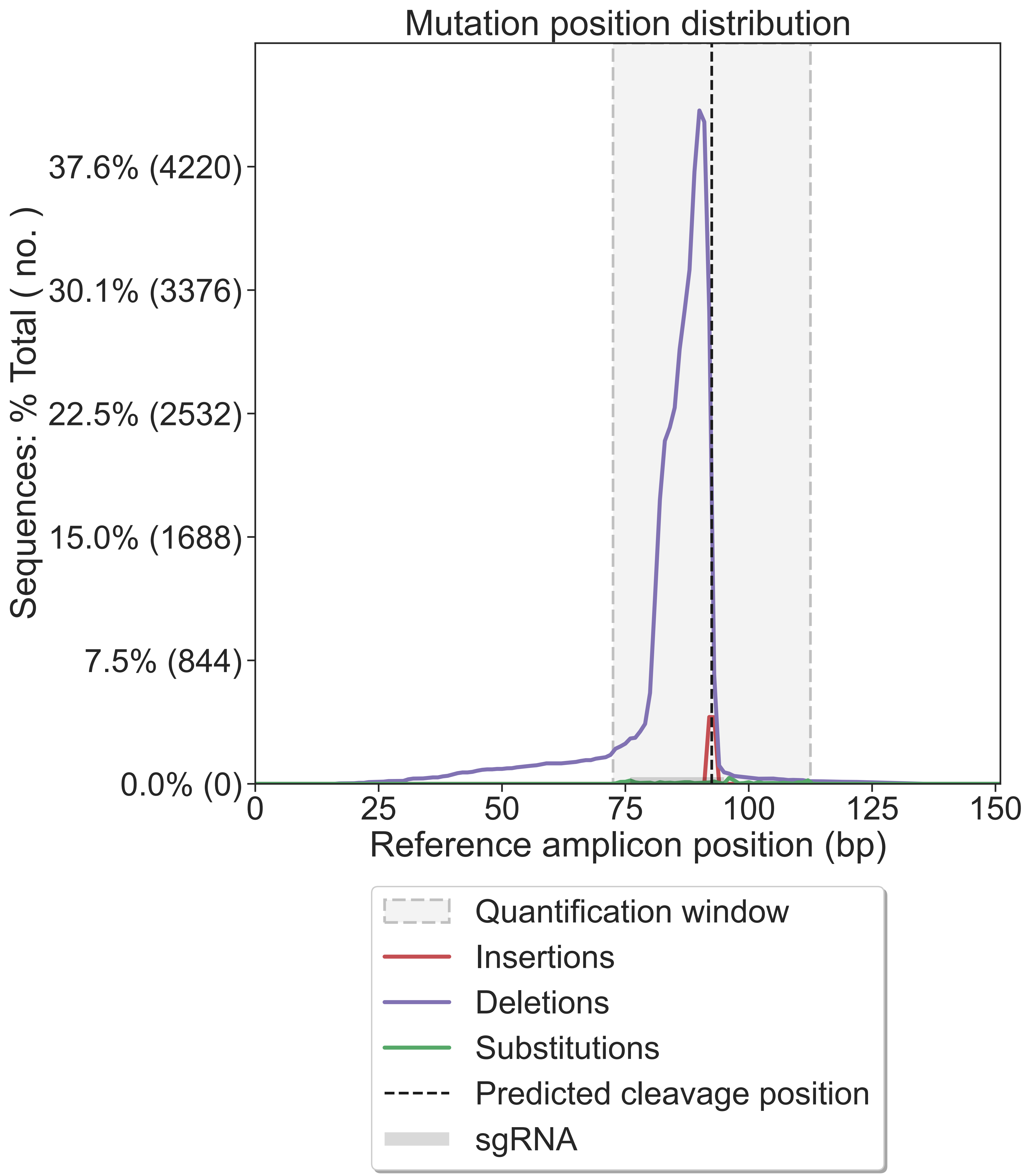

Mutation position distribution
37.6% (4220)
30.1% (3376)
22.5% (2532)
15.0% (1688)
7.5% (844)
0.0% (0)
Sequences: % Total (no.)
0
25
50
75
100
125
150
Reference amplicon position (bp)
Quantification window
Insertions
Deletions
Substitutions
Predicted cleavage position
sgRNA
